# Explanation of Two Well Known Symptoms of Phthisis

**Published:** 1872-06

**Authors:** 


### EXPLANATION OF TWO WELL KNOWN SYMPTOMS OF PHTHISIS.

Dr. F. A. Hartsen writes to the London *Medical Times and Gazette*, November 11, 1871:

Much has been written upon, and still more spoken of, a certain sound—so unwelcome to the physician, so importunate to the invalid by its tick, tick, tick, in the silence of the night. In spite of many hypotheses, the "cliquetis metallique" of lung cavities (for this is the pathological music to which I allude) is enveloped in a cloud of mystery. Some try to explain it as the effect of a drop falling from the top to the bottom of the cavity; others attribute it to the bursting of a bubble.

The first explanation, to my mind, is entirely unsatisfactory. The sound in question is heard in cases even where the fluid contained in the cavity is of a viscous nature. Now, it would be contrary to all rules of accoustics, that a drop of viscous matter—even if the formation of such a drop in a cavity were probable—falling from so slight an elevation upon a viscous surface should produce a ticking sound. As to the second hypothesis, it evidently represents the "metalic sound" as a kind of "rale crepitant," on a large scale. It, however, is insufficient, so long as it has not been proved experimentally that a metalic sound can be produced by a bubble of viscous matter.

This objection cannot be made to a new hypothesis which I would offer to the judgment of competent readers. It has often occurred to me that a sound exhibiting a remarkable analogy to the "cliquetis metallique" may be produced in this manner: two surfaces of a mucous membrane covered by a small quantity of viscous matter are brought into contact, and afterwards separated. The sound is detected the moment of separation. Such will be the case at least, if the said mucous membrane is sufficiently stretched, and has the opportunity of communicating its vibrations to a trumpet-like column of air. So, for example, the metalic sound can be imitated by the surface of the nostrils, and especially by the corners of the lips.

I conclude, by analogy, that most probably this mysterious "cliquetis metallique" is produced by the linings of the cavity losing their hold upon each other, after having been partly brought into contact by the movement of respiration. As no cavity is limited on *all* sides by bony matter, there is always an opportunity for the reconnection of these linings.

Another pathological symptom, of which I wish to discuss the explanation, is the anti-æsthetic hooked appearance of the nails.

Dr. Taylor, if I am not mistaken, attributes this to a process of tuberculization in the roots of the nails. I do not consider this phenomenon of such a serious and complicated origin.

By careful observation, we shall find that the shape of the nails varies with the state of the patient's nutrition. If he regains flesh, the nails gradually recover their normal shape. It seems to me that an abnormal growth of the nails is simply a symptom of emaciation.

The disappearance of the conjunctiva from under the nail deprives it of its natural support. It must henceforth rest almost immediately upon the bone as upon a model, and is obliged to follow the direction of its surface. This surface being rounded at the top, the nail takes a direction downward. If my memory fail not, the late Professor Niemeyer has explained this phenomenon by the absorption of the substance under the nail.

In one case of phthisis under my obse vation I remarked another peculiarity which has been, perhaps, hitheto overlooked. It is this: if you allow the nail to grow, its breadth, far from being the same in all parts, increases gradually toward the top—in fact, the nail assumes a cuneiform or conic shape. This curious and equally unæsthetic phenomenon confirms my explanation of the former; for this is evidently owing to the disappearanee of the supporting conjunctiva.

As the surface of the phalanx bone is more or less flat on the outside, the disappearance of the conjunctiva causes the nail to be flattened. And as the disappearance increases by degrees toward the top of the finger, the flattening of the nail will increase in the same way. Consequently, the nail, from cylindrical, will become conic.

To me, it appears that this nail phenomenon is more apparent in the toe-nail than in the finger-nail. If this be the case, it is in accordance with my (Niemeyer's ?) explanation. For it is to be expected that, the feet being further removed than the hands from the great source of nutrition (the heart), they will more quickly suffer from any decrease of nutrition. In fact, "cold feet" is a more common complaint than "cold hands."

One more remark in conclusion. If this explanation of crooked nails be, as I think, the correct one, it is clear that this phenomenon has no importance whatever as a diagnostic for tubercles, but must occur in every form of emaciation. Observation must decide if this be really the case.

A very rapid growth of the nails and hair seems to belong equally to the symptoms of emaciation. This, however, may be generally known, and I do not attempt to explain it here.—*Half-Yearly Compendium.*

